# Fixation of the mobile fragment in periacetabular osteotomy: a clinical study of two- vs. three-screw fixation with 4-week partial weight bearing

**DOI:** 10.1007/s00402-025-05996-5

**Published:** 2025-08-11

**Authors:** Sufian S. Ahmad, Quentin Karisch, Henning Windhagen, Marco Haertlé

**Affiliations:** Department of Orthopaedic Surgery, Hannover Medical School, Hannover , Germany

**Keywords:** Periacetabular osteotomy; PAO; hip dysplasia; hip preservation; fixation; pelvis; hip arthroscopy

## Abstract

**Aim:**

Fixation of periacetabular osteotomy (PAO) has been a matter of interest since development of the procedure. Despite the stability of the construct, no consensus regarding the minimum number of screws needed for fixation is present. The aim of this study was to compare two-screw and three-screw fixation techniques in the clinical setting.

**Methods:**

The study included a consecutive series of 100 hips that had undergone PAO surgery by a single surgeon between January 2022 and July 2023 with complete radiographic follow-up. The mobile fragment was fixed using three screws in 27 *hips* and two screws in 73 *hips*. Both groups did not significantly differ in any morphometric measure. Lateral center edge angle (LCEA), Acetabular index (AI), extrusion index (EI), anterior wall (AWI), and posterior wall index (PWI) were measured by two independent investigators preoperatively, immediately after surgery and at 1 year follow-up and interobserver agreement measured. Analysis of variance (ANOVA) was used for comparison.

**Results:**

No change of correction of > 4° was observed in any hip in both groups. Both groups did not significantly differ regarding any change in radiographic measure in ΔLCEA (1.09 ± 2.46 vs. 0.52 ± 3.11, *p* = 0.34), ΔEI (− 1.94% ± 2.97% vs. − 0.44% ± 5.75%, *p* = 0.25), ΔAI (− 0.10 ± 1.71° vs. 0.21 ± 1.83, *p* = 0.72), ΔAWI (0.25% ± 7.78% vs. 0.02% ± 12.05%, *p* = 0.57), ΔPWI (4.03% ± 9.94% vs. 3.43% ± 10.79%, *p* = 0.38). There was no difference in complications between groups. The rate of non-union at 1 year was lower in the two screw group, although not significant (7% vs. 17%, *p* = 0.38).

**Conclusion:**

The results emphasize the inherent stability of the PAO construct and demonstrate that the use of two screws for fixation of the mobile fragment is sufficient in PAO surgery, provided that the fragment was fully mobilized during surgery and at least 50% bony contact on the iliac wing was achieved. Furthermore, a 4 week partial weight bearing regimen is also adequate during the healing phase.

## Introduction

Periacetabular osteotomy (PAO) has gained wide recognition as a powerful surgical treatment of dysplastic acetabular morphology [1–4]. One of the main advantages of PAO is its preservation of the integrity of the posterior column, which is critical for maintaining the inherent stability of the construct [2]. The potential clinical implications of increased stability include simpler fixation, which may involve the use of fewer screws, reduced surgical time, a smaller skin incision, and a lower risk of implant-associated complications [5].

In his initial article introducing PAO as a novel procedure, Reinhold Ganz stated that the PAO construct is sufficiently stable to be secured with just two screws. However, the majority of subsequent studies have described the use of a minimum of three screws for fixation of the acetabular fragment [2, 6–8]. Numerous biomechanical studies have evaluated various types of screw fixation techniques. However, there is a paucity of clinical research directly comparing different fixation scenarios [9–11]. To date, only one clinical study has reported on PAO fixation using two screws in a cohort of 32 dysplastic hips [5].

This study aimed to evaluate the radiographic outcomes of two-screw versus three-screw fixation of the acetabular fragment in PAO, specifically focusing on change of correction and union rates. We hypothesized that there would be no significant difference between both fixation methods regarding radiographic change of correction during the first postoperative year.

## Methods

**Fig. 1:**
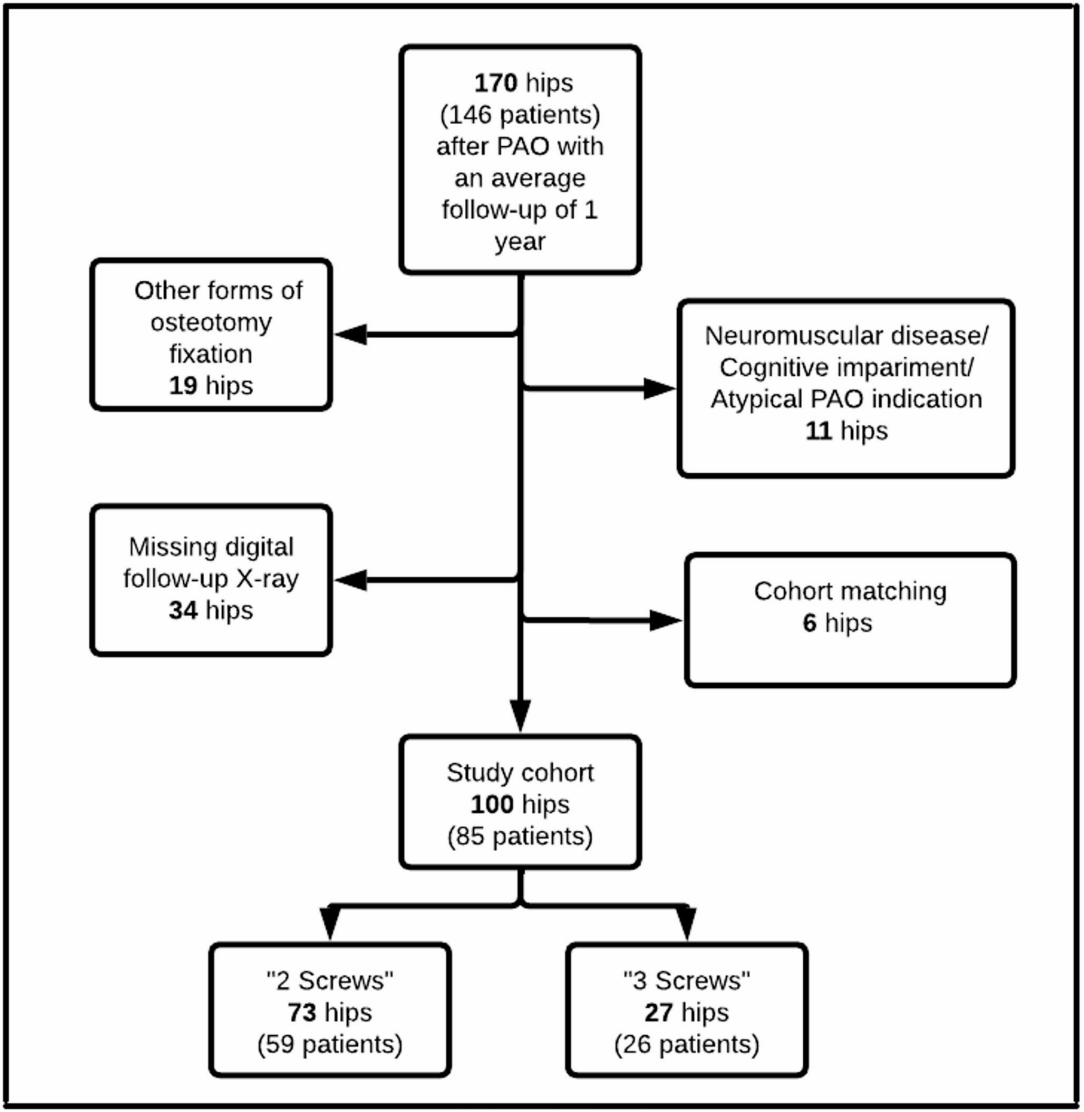
The flowchart highlights the exclusion criteria of this study

The study included a consecutive series of 100 hips in 85 patients that had undergone PAO surgery by a single surgeon between January 2022 and July 2023. All hips that had undergone PAO surgery with the fragment being fixed using either two descending screws or three screws were considered eligible for inclusion in the study, provided that the indication was hip dysplasia with reduced lateral coverage, and there was adequate documentation of radiographic follow-up for an average of 1 year after surgery. Exclusion criteria included PAOs performed for excessive acetabular overcoverage or in patients with neuromuscular disorders (Fig. 1). The decision to use two or three screws to fixate the PAO fragment was made independently of one another, so that both techniques were used independently of one another at the beginning of the retrospective study period.

### Surgical technique

Surgery was performed using a modified Smith-Petersen approach with the patient in the supine position. A 5–10 cm bikini-like incision was made just inferior to the anterior superior iliac spine (ASIS) in line with the inguinal crease. In patients undergoing simultaneous offset correction of the femoral head-neck junction, a longitudinal incision was made starting lateral to the ASIS, extending slightly towards the thigh.

Subcutaneous dissection was performed to identify the blue tensor fascia. The tensor fascia was incised, and the muscle retracted laterally, defining the distal limb of the approach. The proximal limb of the approach was developed 1 cm proximal to the ASIS by incising the abdominal aponeurosis and performing subperiosteal elevation of the iliacus. A Hohmann retractor was then inserted into the subiliac window. Both windows of the approach were connected by subperiosteal medial mobilization of the inguinal ligament and the sartorial insertion from the ASIS medially, exposing the medial hemipelvis and the pubic root.

A blunt scissor was then inserted into the infracapsular region between the rectus and psoas tendons to develop the space for the ischial cut. A Ganz osteotome was then placed on the ischial bone medially. Fluoroscopic images in both anteroposterior and iliac oblique views were taken to ensure an accurate ischial cut towards the ischial spine while protecting the posterior column. This process was repeated from medial to lateral until the entire width of the ischium was osteotomized.

The psoas bursa was then incised over the pubic root, and a spiked Hohmann was hammered into the pubic bone 1 cm medial to the pubic root. After the periosteum was incised, subperiosteal protection of the neurovascular bundle was ensured prior to performing the pubic cut, which was made from lateral to medial starting 5 mm medial to the pubic root. The abductors were incised lateral to the ASIS, and a subperiosteal tunnel was developed towards the greater sciatic notch. The supra-acetabular cut was then made towards the greater sciatic notch, ending approximately 1 cm away from the pelvic brim to ensure the continuity of the posterior column. A straight 15 mm osteotome was then angled towards the ischium, and a straight cut through the bony substance of the posterior column was performed towards the ischial cut. The final bony bridges between the ischial and retroacetabular cuts were fractured using an angled osteotome from inside the pelvis. Finally, two Schanz screws were used for re-orientation under fluoroscopic control. Significant emphasis was placed on fully mobilizing the fragment to allow for tension-free rotation in all directions with minimal effort by the surgeon. This was successfully achieved in all cases.

The correction was carried out by rotating the fragment around the femoral head primarily along a flexion vector, while distributing the bony displacement through elevation of the pubic root and lateral displacement of the iliac wing of the mobile fragment, ensuring at least 50% cortical contact in the region of the supra-acetabular osteotomy after correction. Fluoroscopy was used to validate the correction. 

After achieving the desired correction, three 3.0 mm K-wires are inserted into the mobile fragment to secure the position temporarily. Two of the wires were placed in the trajectory of the later screws. After placement of the wires, final control of accurate correction and wire position was performed prior to fixation. 

Fixation was performed using either two screws or three screws as follows: 

### Two-screw fixation

As mentioned above, two of the K-wires are replaced by screws. This was performed by removing the 3.0 mm K-wire, tapping, and inserting the screw after measurement of the length of the screw.

The following rules were applied for two screw fixation:

1. Full mobility of the fragment verified by the capability of the surgeon to freely rotate the mobile fragment using a Schanz screw as a joystick with one hand, and the fragment remains in place after correction.
2. One descending screw from the iliac crest placed medially in the bony substance of the pelvic brim in close proximity to the pubic root.
3. One descending screw placed laterally in the anterior column, in the bony substance of the anterior inferior iliac spine (AIIS).
4. At least 7 mm of bony purchase on either side of the screw, meaning a minimum of 28 mm bone purchase of both screws.

### Three-screw fixation

The following rules were applied for three screw fixation.

5. Rules 1–4 were applied.
6. A third screw was inserted, either descending as described above, or horizontally ascending, starting from the AIIS of the mobile fragment and ending in the posterior column of the stable pelvis.

Weight-bearing was restricted to 15 kg for the initial 4-week period, with knee flexion limited to 90 degrees. Subsequent to this phase, patients were allowed to progress to full weight-bearing as tolerated. Participation in contact sports was permitted after a minimum duration of 3 months.

### Clinical and radiographic outcome

Demographic data including age, gender and BMI were tabulated. All patients received standardized conventional antero-posterior (AP) X-rays prior to surgery, immediately after surgery during the hospital stay, at 4–6 weeks follow-up and 12 months [12].

Hip morphometric measures were measured prior to surgery at each follow-up by two independent investigators. The measures angles included the lateral center edge angle (LCEA), acetabular index (AI), extrusion index (EI), anterior wall index (AWI) and posterior wall index (PWI) [13].

Revisions of any form, delayed unions, loss of correction defined as a change in LCEA of more than 4°, and implant failure, were noted [9].

### Statistical analysis

Results were tabulated and presented as means (± standard deviation). Events and complications were presented as frequencies. The interrater reliability was calculated for radiographic measures using the Intraclass Correlation Coefficients (ICC). Additionally, interrater reliability was illustrated using a Bland-Altman plot. Power analysis revealed the need of 25 hips per group to determine a clinically relevant change in correction of 4°, given an alpha of 0.05 and power of 0.8.

Comparison between means was performed using analysis of variance (ANOVA). SPSS IBM statistics was used for analysis.

## Results

In this study, a total of 100 PAOs were analyzed. Two screws were used to fixate the pelvic osteotomy in 73 cases (73.00%), while three screws were used in 27 cases (27.00%). Both cohorts did not significantly differ in terms of preoperative radiographic features of hip dysplasia (Table 1).

**Table 1:** Descriptive data “two-screw fixation” vs. “three-screw fixation”

Number of hips	2 screw fixation	3 screw fixation	*p*-value
	73/100 (73.00%)	27/100 (27.00%)	
Ø Age (years)	29.01 ± 8.235	27.19 ± 7.771	0.492
Male	20/73 (27.40%)	5/27 (18.52%)	0.443
Body mass index (kg/m2)	24.08 ± 4.27	24.88 ± 5.16	0.44
Left hip	32/73 (43.84%)	11/27 (40.74%)	0.824
Preoperative LCEA (°)	18.48 ± 4.83	17.81 ± 6.72	0.173
Preoperative acetabular index (°)	12.91 ± 4.54	13.49 ± 5.78	0.597
Preoperative extrusion index	27.13% ± 5.26%	28.17% ± 6.29%	0.255
Preoperative anterior wall index	38.29% ± 11.55%	41.10% ± 17.65%	0.849
Preoperative posterior wall index	84.74% ± 15.57%	76.74% ± 22.54%	0.139

**Fig. 2:**
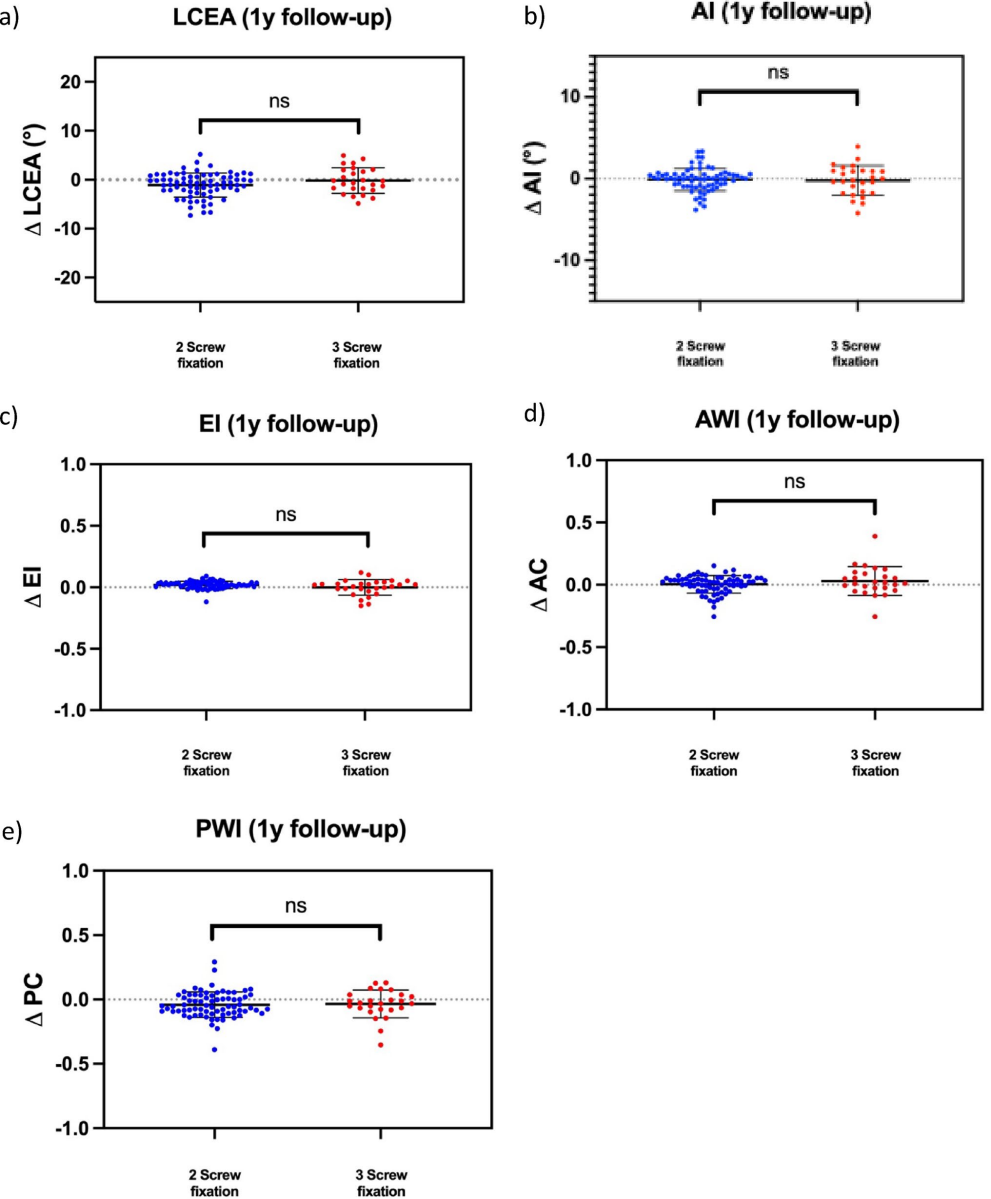
**a** At the 1year postoperative followup, patients who underwent PAO with two-screw fixation demonstrated a mean change in the lateral center–edge angle (LCEA) of 1.09° ± 2.46°. Those treated with threes-crew fixation showed a mean LCEA change of 0.52° ± 3.11° (*p* = 0.341). **b** The mean change in the acetabular index (AI) with two-screw fixation was − 0.10 ± 1.71°. In contrast, the Delta value of the AI with three screw fixation was 0.21 ± 1.83 (*p* = 0.718). **c** With two screws in place, the average change in the extrusion index (EI) 12 months postoperatively was − 1.94% ± 2.97%. In contrast, fixation with three screws resulted in an average change in AI of − 0.44%±5.75% (*p* = 0.255) within one year after PAO. **d** The postoperative anterior wall index (AWI) demonstrated no significant change in the postoperative course independent of the fixation technique (two screws 0.25% ± 7.78% vs. three screws 0.02% ± 12.05; *p* = 0.569). **e** In the postoperative course after PAO, the posterior wall index (PWI) showed an average change of 4.03% ± 9.94% in patients with two-screwfixation of the osteotomy. In contrast, there was a PWI change of 3.43% ± 10.79% with fixation using three screws (*p* = 0.382)

The postoperative measurements of LCEA, AI, EI, AWI, PWI showed no change of correction when comparing the immediate postoperative X-ray with the 1-year follow-up X-ray, regardless of the number of screws used (Fig. 2; Table 2).

**Table 2:** Postoperative radiographic follow-up “two-screw fixation” vs. “three-screw fixation”

(a)				
	LCEA (°) -postoperative-	LCEA (°) -1 y follow up-	Δ LCEA (°)	p-value
two-screw fixation	32.55 ± 5.88	33.64 ± 6.82	1.09 ± 2.46	0.341
three-screw fixation	30.89 ± 5.75	31.41 ± 7.00	0.52 ± 3.11	

(b)				
	AI (°) -postoperative-	AI (°) -1 y follow up-	Δ AI (°)	p-value
two-screw fixation	0.84 ± 5.10	0.74 ± 5.24	-0.10 ± 1.71	0.718
three-screw fixation	2.79 ± 6.16	3.00 ± 6.62	0.21 ± 1.83	

(c)				
	EI -postoperative-	EI -1 y follow up-	Δ EI	p-value
two-screw fixation	14.95% ± 5.50%	13.01% ± 5.85%	-1.94% ± 2.97%	0.255
three-screw fixation	14.78% ± 6.51%	14.34% ± 7.69%	-0.44% ± 5.75%	

(d)				
	AWI -postoperative-	AWI -1 y follow up-	Δ AWI	p-value
two-screw fixation	40.50% ± 17.08%	39.49% ± 14.98%	0.25% ± 7.78%	0.569
three-screw fixation	40.22% ± 18.65%	40.29% ± 19.44%	0.02% ± 12.05%	

(e)				
	PWI -postoperative-	PWI -1 y follow up-	Δ PWI	p-value
two-screw fixation	84.52% ± 17.77%	88.55% ± 19.87%	4.03% ± 9.94%	0.382
three-screw fixation	78.54% ± 23.42%	82.87% ± 24.59%	3.43% ± 10.79%	

Table 2 displays the absolute measured values of LCEA, AI, EI, AWI and PWI of both study groups postoperatively and 1 year after surgery. In addition, the delta value of the absolute change in measured values of the corresponding parameters is shown. The P-value given refers to the significance of the two delta values when comparing the two study groups

In both study groups, there was no postoperative corrective change of > 4° in the one-year interval (Table 3).

**Table 3:** Incidence of secondary outcome endpoints “two-screw fixation” vs. “three-screw fixation”

	two-screw fixation	three-screw fixation	*p*-value
Non-union	5/66 (7.58%)	4/23 (17.39%)	0.229
Implant failure	2/66 (3.03%)	1/23 (4.35%)	1.000
Change of LCEA correction > 4°	0/73 (0.00%)	0/27 (0.00%)	

**Fig. 3:**
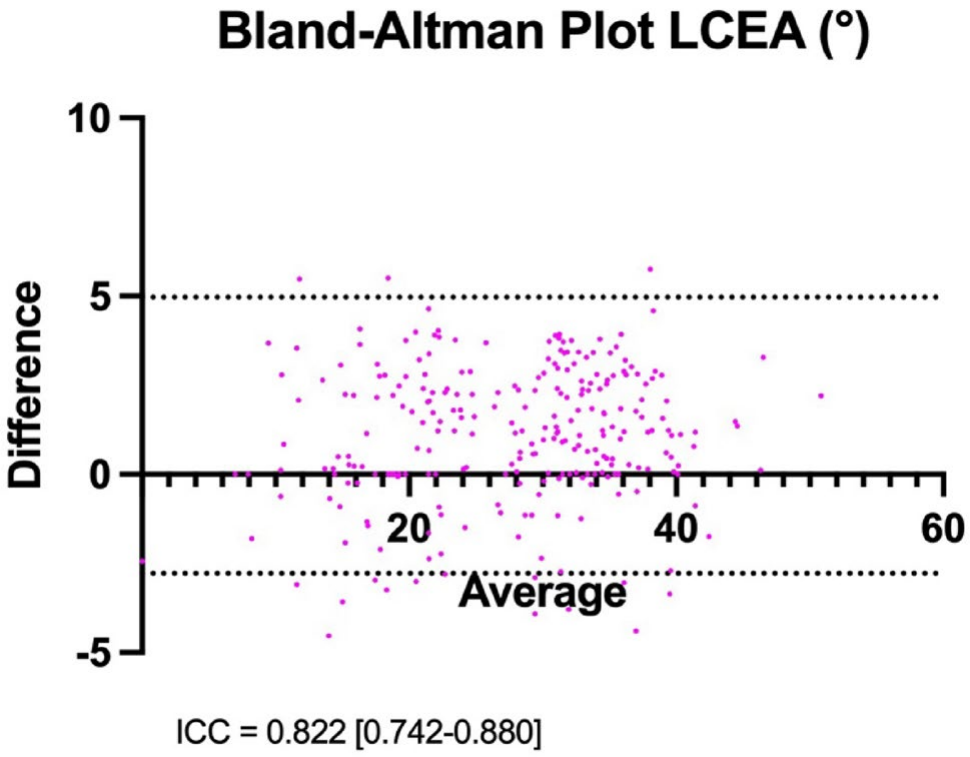
An interclass class coefficient (ICC) of 0.822 [0.742–0.880] was calculated for two independent

The inter-rater reliability calculation indicated a strong correlation in radiographic measurements between both investigators (Fig. 3).

Regarding the incidence of non-unions and implant failures, no significant difference was observed between the two cohorts (Table 3). All of the observed implant failures occurred in the interval between 6 weeks and 12 months after surgery. No screw breakage was observed in the period of 6 weeks after surgery. None of these cases showed a significant loss of correction compared to the immediate postoperative results. All observed cases of non-union occurred in the region of the pubic osteotomy. In all cases of implant failure, only one of the two or three screws broke. With respect to implant-associated complications or pubic non-unions, no revision surgery was required. No instances of acetabular fractures, particularly involving the posterior column, were reported in either cohort.

## Discussion

**Fig. 4:**
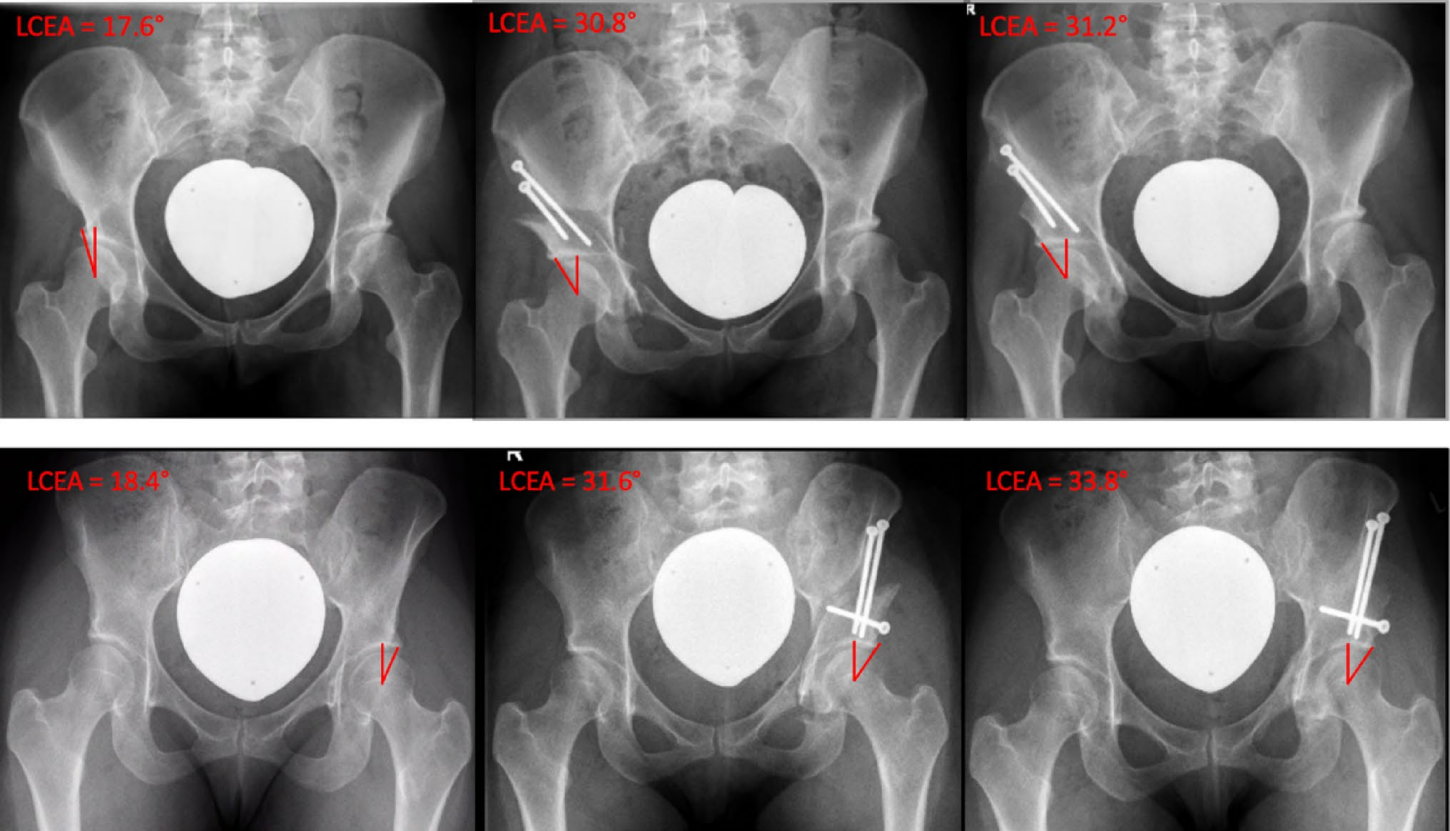
**a** The X-ray images show a sufficient correction of the undercoverage of the hip with two-screw fixation of the osteotomy fragment. The postoperative course demonstrates no significant change in the result of the correction one year after PAO. **b** In comparison, fixation of the acetabular PAO fragment with three screws also showed no significant change in the LCEA in the postoperative course up to one year postoperatively. The bony consolidation of the osteotomy gaps was also completed and comparable to the findings in (**a**)

The most important finding of this study was that there was no significant difference in whether two or three screws were used for fixation of the mobile fragment in PAO, in the clinical setting, provided that the rules mentioned above were applied. Furthermore, a 4-week 10 kg partial weight-bearing protocol is sufficient (Fig. 4).

The results may appear unexpected, especially when considering biomechanical studies that emphasize the importance of a third or even fourth screw in maintaining construct stability [10, 14, 15]. However, the in-vivo scenario differs in several critical aspects, most notably due to the frequent neglect of the pelvi-femoral musculature in biomechanical studies. Not to depreciate the value of in-vitro biomechanical studies but as an explanatory concept as to why two screws are enough in clinical practice.

The combined action of the pelvi-trochanteric muscles and abductors on the femur, along with the natural tension between the femoral head and acetabular cartilage, generates a cranial vector that stabilizes the mobile fragment. This enhances the stabilization of the interlocked mobile fragment by compressing the contact points on the iliac, ischial, and pubic regions. The two screws used in this procedure primarily serve to control rotation. Consequently, our findings corroborate the initial conclusions drawn by Reinhold Ganz in his seminal 1988 publication on PAO [2].

The implications of these results are particularly significant for patients undergoing minimally invasive PAO. The potential necessity for a third screw may require extending release of the abdominal aponeurosis from the iliac crest, especially in smaller pelvises. Furthermore, screw removal is much more straightforward and would require less dissection and surgical time.

Most importantly, it is relevant for surgeons to recognize that, while the third screw can enhance stability, it is not essential and can be oriented in various directions based on the individual anatomy.

The rate of complications and implant failure did not differ between the two groups of two- vs. three screws. Interestingly, the absolute rate of delayed union at any of the osteotomy sites was lower in the group fixed with two screws, though not significant. This may be of interest, given that it has been previously reported that less rigid forms of fixation allow for better healing of the osteotomy and fewer non-unions [9, 16].

Søballe reported on the use of two screws for fixation in minimally invasive PAO and published the results of radiometric analysis in a series of 32 dysplastic hips that had undergone PAO [5, 17]. The patients were allowed full weight bearing after 8 weeks.

The results of the current study confirm these findings. As a matter of fact, full weight bearing was allowed after 4 weeks, rather than 8 weeks in all patients. This further confirms that earlier weight bearing is also possible with two screws.

Another technical feature should be emphasized at this point. In all PAOs, it was verified that the PAO fragment was fully mobile after the osteotomy, as our clinical experience showed that additional tension forces made it difficult to hold the PAO fragment in position after correction. If a full release of the PAO fragment was achieved, fixation with two or three screws could keep the correction. However, this is a retrospective clinical observation by the investigators. Based on the current literature, further biomechanical studies are required to investigate this effect.

The limitations of this cohort study is the non-randomized design which results in a reduced level of evidence because a selection bias cannot be ruled out. However, both groups were comparable in all measures in order to ensure comparability. A further limitation is that RSA was not used. However, morphometric radiographic measures were performed by individual investigators as done in actual clinical practice. The interobserver reliability in this study was comparable to the literature [18, 19].

The findings of this study indicate that the application of two screws for the fixation of the mobile fragment inPAO is safe, provided that the fragment remains fully mobile during surgery and is free of tension. Furthermore, a 4-week partial weight-bearing regimen was sufficient to maintain stability during the healing phase using two screws. Emphasis should be placed on ensuring the acetabular fragment is fully mobilized before reorientation, as this would allow for less complex fixation. These results have significant implications for minimally invasive PAO procedures, as the use of additional screws could increase both the length of the surgical incision and the overall surgical time.

However, both screws must achieve adequate bony purchase and fixation. In case of uncertainty, the authors recommend using three screws.

## Data Availability

No datasets were generated or analysed during the current study.
